# The digital divide in rural and regional communities: a survey on the use of digital health technology and implications for supporting technology use

**DOI:** 10.1186/s13104-024-06687-x

**Published:** 2024-03-28

**Authors:** Hannah Jongebloed, Kate Anderson, Natalie Winter, Lemai Nguyen, Catherine E. Huggins, Feby Savira, Paul Cooper, Eva Yuen, Anna Peeters, Bodil Rasmussen, Sandeep Reddy, Sarah Crowe, Rahul Bhoyroo, Imran Muhammad, Anna Ugalde

**Affiliations:** 1 Institute for Health Transformation, Deakin University, 1 Gheringhap Street, Geelong, 3220 Australia; 2 School of Nursing & Midwifery, Faculty of Health, Deakin University, Geelong, Australia; 3 School of Computing Technologies, STEM College, RMIT University, Melbourne, Australia; 4 School of Health & Social Development, Faculty of Health, Deakin University, Geelong, Australia; 5 Deakin Business School, Faculty of Business and Law, Deakin University, Geelong, Australia; 6 School of Medicine, Faculty of Health, Deakin University, Geelong, Australia; 7 Department of Public Health, Faculty of Health and Medical Sciences, University of Copenhagen, Copenhagen, Denmark; 8 Faculty of Health Sciences, University of Southern Denmark, Odense, Denmark; 9 Western Victoria Primary Health Network, Geelong, Australia

**Keywords:** Digital divide, Digital health, Confidence, Rural health, Online health information, Accessibility, Digital literacy

## Abstract

**Objective:**

A digital divide exists for people from rural and regional areas where they are less likely and confident to engage in digital health technologies. The aim of this study was to evaluate the digital health literacy and engagement of people from rural and regional communities, with a focus on identifying barriers and facilitators to using technology.

**Results:**

Forty adults living in rural/regional areas completed a survey consisting of the eHealth Literacy Scale (eHEALS) with additional items surveying participants’ experience with a range of digital health technologies. All participants had used at least one digital health technology. Most (80%) participants had an eHEALS score of 26 or above indicating confidence in online health information. Commonly reported barriers to digital health technology use centred on product complexity and reliability, awareness of resources, lack of trust, and cost. Effective digital health technology use is becoming increasingly important, there may be a need to prioritise and support people with lower levels of digital health literacy. We present opportunities to support community members in using and accessing digital health technology.

## Introduction

Health literacy relates to a person’s capacity to find, understand, use, and critically appraise health information [1]. Health resources have quickly expanded into the digital domain as digital health tools, technologies and products are increasingly necessary to navigate medical information and health care [2]. Digital health literacy refers to seeking, finding, understanding, and appraising electronic sources of health information to manage one’s own health [3]. Maintaining a sufficient level of health literacy is needed to support and empower people to utilise digital tools to manage their health. This is associated with a range of health management and behavioural outcomes [4].

The digital divide refers to the gap in access and use of technology between individuals, households, and countries. With an increasing dependence on digital health services since the recent pandemic, people without health access are increasingly disadvantaged and COVID-19 has further exacerbated digital inequities. The digital divide experienced in rural areas continues to exist [5]. In addition to infrastructure barriers, demographic trends in rural and regional areas (including older age, lower income, and lower formal education levels) are also associated with lower digital health [5, 6]. Despite this, rural populations have demonstrated a desire to engage with digital health technologies [7]. There is a need to understand how rural and regional communities engage with digital health technologies in order to consider how technology use can be supported in these communities to reduce the digital divide.

The aim of this study was to evaluate the digital health literacy and engagement of people from rural and regional communities, with a focus on identifying barriers and facilitators to using technology.

## Methods

We recruited people from the general community living in a rural or regional area in Victoria, Australia. Participants were part of Western Victoria Primary Health Network’s (WVPHN) catchment area; an area over 79,000 square kilometres, including towns Ballarat, Geelong, Horsham and Warrnambool. This region has a population of over 620,000 (approximately 32% of regional Victoria’s population) [8, 9]. Participants were adults (18 years and older) who could read, write, and understand English.

Participants were recruited via paid advertising on Facebook. Flyers with the study information and QR code to the survey were also disseminated across the community, in settings such as local general practice and specialist and community health clinics.

Participants completed an online survey which included demographics (e.g., age, gender, country of birth, local government area in which they reside, education level, and employment status). The eHealth Literacy Scale (eHEALS) [10] was administered to measure digital health literacy. The eHEALS consists of eight items, with a 5-point Likert scale, ranging from “strongly disagree” to “strongly agree”. Two further items included in the eHEALS assessed participants perspectives regarding the usefulness and importance of digital health but did not contribute to participants total eHealth literacy score.

In addition to the eHEALS, a multiple choice item was included to survey the range of digital health products used. Three open-ended questions were used to identify facilitators of and barriers to digital health use and additional support to support digital health engagement. Descriptive analysis using Microsoft Excel software was conducted. Total eHealth literacy scores range from 8 to 40; with scores 26 and above indicating high eHealth literacy and < 26 indicating low eHealth literacy [11–13]. For the open-ended questions, responses were coded into common categories and the frequency of responses were then tallied within each category.

## Results

Forty participants completed the survey. The mean age was 46.2 years (SD = 15.1, range = 24–82), most were born in Australia (*n* = 33; 83%) and approximately half resided in the greater Geelong area (*n* = 21; 53%). Sample characteristics are provided in Table 1.

**Table 1:** Participant characteristics

Variables	N (%)
Gender	
Female	26 (65)
Male	14 (35)
Country of Birth	
Australia	33 (82.5)
Other	7 (17.5)
Local Government Area	
Greater Geelong City	21 (52.5)
Ballarat City	8 (20)
Surf Coast Shire	3 (7.5)
Corangamite Shire	2 (5)
Northern Grampians Shire	1 (2.5)
Southern Grampians Shire	1 (2.5)
Glenelg Shire	1 (2.5)
Horsham Rural City	1 (2.5)
Warrnambool City	1 (2.5)
Moorabool Shire	1 (2.5)
Education Level	
Postgraduate degree	15 (37.5)
University Degree	12 (30)
TAFE course or diploma	8 (20)
Some of high school	5 (12.5)
Employment Status	
Working full time	22 (55)
Working part time	8 (20)
Retired	4 (10)
Home or caring duties	2 (5)
Other	2 (5)
Working casual hours	1 (2.5)
Out of work and looking for work	1 (2.5)

### Digital health literacy

The average score on the eHEALS was 30 (SD = 6.4, range = 8–40). Thirty-two participants (80%) had an eHEALS score of 26 or above, falling within the high literacy range and eight (20%) had a score of 25 or lower, indicating low health literacy.

Two additional questions from the eHEALS showed that the majority of participants (*n* = 33; 83%) found the internet to be useful or very useful in making decisions about their health. The majority (*n* = 35; 88%) also considered it to be important or very important to have access to health resources via the internet.

**Fig. 1:**
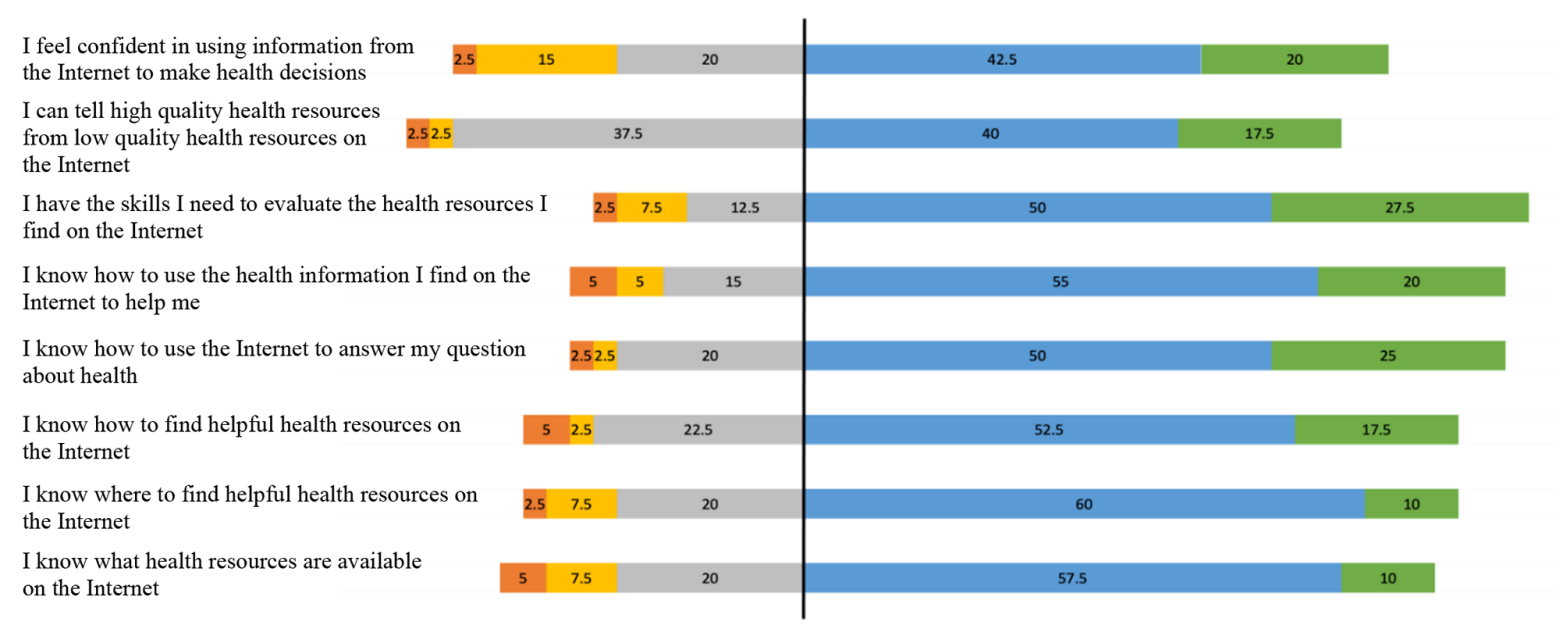
Responses as percentages for eHEALS questionnaire items

Each eHEALS item and the response frequencies (as a percentage) are shown in Figure 1. Most participants (58%– 78%) endorsed either agree or strongly agree for each of the 8 items, reflecting the high digital health literacy of the sample. Whilst 58% of participants (n = 22) agreed or strongly agreed with the statement: *I can tell high quality health resources from low quality health resources on the Internet* (item 9), 38% (n = 15) responded as ‘undecided’. This is the highest proportion of undecided responses of the 7 items (range: 13%–23%).

### Engagement with digital health products

**Fig. 2:**
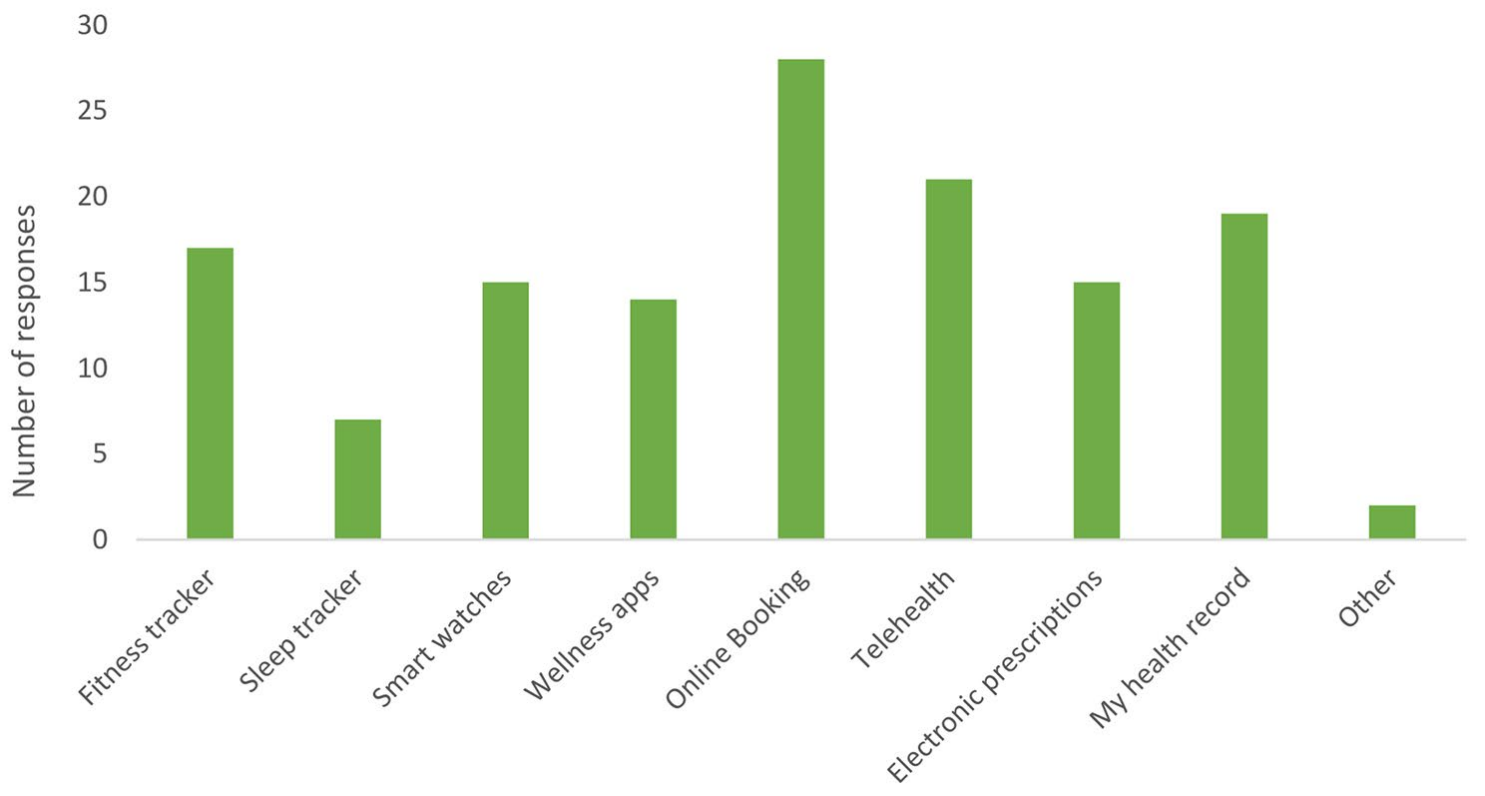
Digital health products most used by participants

Participants identified digital health products they used most frequently. All participants identified having used at least one digital health product, with 85% identifying more than one product. Two participants identified ‘Other’ products including the organ donation register and gym applications. Figure 2 displays the number of respondents that reported using each product.

Three open ended questions were utilised to understand: (1) what makes it easier to use digital health technologies, (2) what makes it harder to use digital health technologies, and (3) how to promote engagement with digital health technologies.

***Facilitators to digital health***: 32 of 40 (80%) valid responses from participants were coded into four categories: convenience, comprehension, trust and interoperability. Most commonly (*n* = 14, 44%), people reflected on the importance of functionality, citing integration, accessibility and convenience as factors. Others reflected the need for comprehensible information and education about digital health technologies (*n* = 4, 13%). Two participants reported that trust in the products was important. Interoperability of digital health technology (between apps, and between users and healthcare providers) was noted by two people.

***Barriers to using digital health***: Twenty-three participants (58%) identified barriers that made it harder for them to access digital health technologies. These were coded into four categories: product complexity, unreliable products, lack of trust in or awareness of products, and cost. Complexity and the lack of reliability (e.g., poorly functioning apps or poor internet connectivity) was the most common barrier, identified by 10 participants. The second most common barrier was unawareness of resources, with six participants reporting issues in selecting digital health technologies. Four people reported a lack of trust in digital health technologies, and three people identified costs as a barrier.

***Promoting engagement***: Survey respondents were asked to comment on additional support and information that would help them engage with digital health products. Eleven people responded (28%) and responses were coded into five themes: information about products, endorsement, clear language, health professional support and community training. Four people noted that better information about products would help with identifying appropriate digital health technologies, and two people suggested endorsement as an appropriate way to establish authenticity and trust in a digital health product. Two people suggested health professionals could assist with developing digital health literacy. Two other respondents noted that the availability of language in a clear format could assist. Another person noted there was opportunity to be shown the products by community groups.

## Discussion

This study advances the current understanding of digital health technology use in regional and rural communities. This sample had relatively high digital health literacy with 80% of the sample scoring high on the eHEALS. All participants have or continue to use at least one digital health technology. Digital health technologies intended to foster engagement with health care services were the most commonly used technologies, including online bookings and telehealth.

Trust was both a facilitator and a barrier to engaging with digital health technologies, whereby higher levels of trust reflected intent to use digital health technologies. Community members have reported being overwhelmed by digital health choices, which may be remedied through discussions with clinicians to assess usability preferences and support informed decision making [14]. Interoperability was valued; however, the development of interoperable systems faces challenges including cost, privacy and variation in coding [15]. Nonetheless, these results suggested that interoperability could be a facilitator to using digital health technologies. Improving the interoperability across platforms while ensuring standards are met [16] is likely to be worthwhile.

### Limitations

This study is limited by the small sample size. The online component of recruitment may also have led to a biased sample given the digital health focus of this work. It is recommended future research focuses on targeting and engaging groups with lower levels of digital health literacy. However, the strategies identified highlight how it might be possible to engage people in rural settings and bridge the digital divide between rural and metropolitan populations. Future work could focus on a qualitative approach to generate a more in depth understanding of digital health use across rural and regional groups. The differences between remote, rural and regional groups in using digital health technologies may be substantial and future work could explore this rather than combine non-metropolitan groups as we did in this study.

Effective digital health technology use is becoming increasingly important and there may be a need to prioritise and support people with lower levels of digital health literacy. There are opportunities for clinician endorsement, additional information and training to support all community members in accessing and using digital health technologies.

## Data Availability

The datasets used and/or analysed during the current study are available from the corresponding author on reasonable request.

## List of abbreviations

WVPHN: Western Victoria Primary Health Network
eHEALS: eHealth Literacy Scale
